# Tumour: Fibroblast Interactions Promote Invadopodia-Mediated Migration and Invasion in Oral Squamous Cell Carcinoma

**DOI:** 10.1155/2022/5277440

**Published:** 2022-11-26

**Authors:** Koroku Kato, Hiroki Miyazawa, Shuichi Kawashiri, Daniel W. Lambert

**Affiliations:** ^1^School of Clinical Dentistry, University of Sheffield, Sheffield S10 2TA, UK; ^2^Department of Oral and Maxillofacial Surgery, Kanazawa University Graduate School of Medical Science, 13-1 Takara Machi, Kanazawa 9208641, Japan

## Abstract

**Objectives:**

In the progression of cancer, interactions between cancer cells and cancer-associated fibroblasts (CAFs) play important roles. Cancer cell invasion is facilitated by filamentous actin (F-actin)-rich membrane protrusions called invadopodia, and the relationship between CAFs and invadopodia has been unclear. We used oral squamous cell carcinoma (OSCC) to investigate CAFs' effects on the formation of invadopodia, and we assessed the expressions of invadopodia markers and CAF markers ex vivo and their relationship with clinical parameters and survival.

**Materials and Methods:**

We examined the effect of culture with normal oral fibroblast (NOF)-derived and CAF-derived conditioned medium on the migration and invasion of two OSCC-derived cell lines using Transwells in the absence/presence of Matrigel. Immunoblotting and immunocytochemistry were conducted to assess the expressions of the invadopodia markers tyrosine kinase substrate 5 (Tks5) and membrane type 1 matrix metalloproteinase (MT1-MMP). We also used immunohistochemistry to examine patients with OSCC for an evaluation of the relationship between the CAF marker alpha smooth muscle actin (*α*SMA) and the expression of Tks5. The patients' survival was also assessed.

**Results:**

Compared to the use of culture medium alone, NOF-CM and CAF-CM both significantly increased the OSCC cells' migration and invasion (*p* < 0.05), and they significantly increased the expressions of both Tks5 and MT1-MMP. After the depletion of Tks5, the OSCC cells' migration and invasion abilities decreased. The expression of Tks5 and that of *α*SMA were correlated with poor survival, and a high expression of both markers was associated with an especially poor prognosis.

**Conclusions:**

These results indicate that the formation of invadopodia is (i) important for OSCC cells' migration and invasion and (ii) regulated by the interaction of OSCC cells and stromal fibroblasts.

## 1. Introduction

In individuals with cancer, the major cause of mortality is the spread of malignant cancer cells to local and/or distant sites. The cancer cells must invade the surrounding tissue and enter the vasculature and/or lymphatics in order to disseminate from the primary site. Invadopodia support cancer cells' invasion into surrounding tissue; the invadopodia are filamentous membrane protrusions formed by invasion cancer cells, and the invadopodia contribute to the remodeling of the extracellular matrix (ECM). Our understanding of the molecular mechanisms underlying the formation of invadopodia has expanded with recent research. For example, it was revealed that two actins (F-actin and *β*-actin) are co-localized at invadopodia, and when both of these forms of actin are overexpressed, the size and number of invadopodia are increased [1]. Invadopodia are enriched with a variety of signaling molecules and membrane remodeling proteins, and invadopodia can also degrade the ECM [2–4]. Many types of cancer cells (including those derived from tumours of the colon, pancreas, head and neck, breast, and prostate) contain invadopodia [5], indicating that the majority of cancer types may tend to form invadopodia [6]. Although important roles in the metastatic cascade have been suggested for invadopodia, the question of how invadopodia are formed remains to be answered.

Invadopodia are known to have a proteolytic function that is dependent on interactions of cell adhesion, cell signaling, adaptor, and actin regulatory proteins in an underlying network [7–9]. The Src substrate tyrosine kinase substrate 5 (Tks5, also known as SH3PXD2A) is an adaptor protein that was observed to regulate ECM remodeling via the modulation of specialized adhesion structures called “podosomes” in normal cell types and “invadopodia” in cancer cells [10, 11]. Tks5 is apparently localized exclusively in cancer cells' invadopodia, and it has been reported that in several human cancer cell lines, Tks5 is required for both the formation of invadopodia and the invasive behavior of invadopodia [12].

The tumour microenvironment has critical roles in the invasion and metastasis of cancer cells [13]. Cancer cells are able to corrupt adjacent stroma to form a permissive, supportive environment for a tumour's progression is formed by stroma that are adjacent to and corrupted by cancer cells. This is made possible by the production of a variety of proteases and protease inhibitors, growth factors, and ECM components by the cancer cells. Cancer cells also activate stromal fibroblasts, enabling them to become cancer-associated fibroblasts (CAFs), at least in part by secreting molecules (e.g., tumour growth factor-beta [TGF-*β*]) that invoke phenotypic changes such as myofibroblastic differentiation and senescence [14–16]. CAFs can promote cancer cell survival [17], growth [18], drug resistance, and both the invasion and the metastasis of cancer cells [19]. CAFs have also shown various effects on the surrounding stroma, including the alteration of the composition and structure of the ECM, the induction of an inflammatory response, and the promotion of angiogenesis. Cancer cells' and fibroblasts' interaction in a tumour microenvironment contributes to the initiation, progression, and metastasis of many types of cancer [20, 21].

Although it is thought that CAFs promote the invasion and metastasis of oral squamous cell carcinoma (OSCC) cells, the ability of CAFs to affect invadopodia formation by OSCC cells remains to be determined. We conducted the present investigation to evaluate the effect of CAFs on invadopodia formation and cancer cell behavior.

## 2. Materials and Methods

### 2.1. Cell Culture

Two OSCC-derived cell lines, SCC4 and H357, and normal oral fibroblasts (NOFs) were cultured in Dulbecco's Modified Eagle Medium (DMEM) with 2 mM L-glutamine and 10% (v/v) fetal bovine serum (FBS). *In vivo*, the cell line SCC4 is highly invasive and H357 is nonmetastatic [22]. The NOFs used in the present study were obtained from patients who underwent the extraction of wisdom teeth, with their informed consent (Sheffield Research Committee approval no. 09/H1308/66) [23].

### 2.2. Generation of Myofibroblastic CAFs

NOF had grown to 70%–80% confluence. The medium was then aspirated from the cultures, and the remaining cells were first washed with Dulbecco's phosphate-buffered saline (PBS), and then, incubated (24 h) in serum-free medium (SFM). Twenty-four hours later, the cells were treated with TGF-*β*1 (R&D Systems, Minneapolis, MN) (5 ng/ml) in SFM for 48 h to induce a cancer-associated fibroblast (CAF)-like phenotype. After 48 h, the medium was changed to fresh SFM, and it was maintained for 72 h before it was collected for use as the conditioned medium (CM).

### 2.3. Culture of Cancer Cells with Normal Fibroblast or CAF Condition Medium

SCC4 cells and H357 cells were each cultured in DMEM containing 10% FBS. When the cells reached 70–80% confluence, the growth medium was removed and replaced with NOF-derived conditioned medium (NOF-CM) or CAF-derived conditioned medium (CAF-CM) for 24 h, and total RNA or cell lysates were then collected.

### 2.4. Immunofluorescence Staining of Cultured Cells

H357 and SCC4 cells were separately seeded on 13-mm-dia. Coverslips made of borosilicate glass (VWR International, Radnor, PA) in 24-well plates and incubated for 24 h. Twenty-four h later, we fixed the cells with 4% formaldehyde diluted in PBS for 15 min at room temperature (RT) and washed them in PBS (5 min) before permeabilisation in 0.2% Triton X-100 in fresh PBS. The nonspecific protein binding was blocked with the use of normal goat serum. Primary antibodies to Tks5 (1 : 10, Proteintech, Chicago, IL), and *β*-actin (1 : 1000, Sigma, St. Louis, MO) were added in normal serum overnight at 4°C. A fluorochrome-conjugated secondary antibody (FITC; Thermo Fisher Scientific, Waltham, MA), diluted in antibody dilution buffer, was added for 1 h at RT in the dark. The coverslips were then washed and mounted on microscope slides with ProLong™ Gold Antifade Reagent, with DAPI (Thermo Fisher Scientific). The cells were then observed by microscopy (Axiovert 200M, Zeiss, Jena, Germany) and AxiVision software (Zeiss).

For the quantitative analysis of the number of invadopodia, three areas were randomly selected and analysed using ImageJ public-domain software. For each condition, the number of Tks5-positive spots per cancer cell was counted.

### 2.5. Short Interfering (si) RNA and Transfection

The reduction of the expression of the marker Tks5 in OSCC cells was achieved with the use of short interfering (si) RNA. The nontargeting control siRNA and the Tks5 siRNA (cat. No. 4392420) were both obtained from Thermo Fisher Scientific (UK). SCC4 cells at 50%–70% confluence were transfected with Tks5 siRNA or non-targeting control siRNA in six-well plates using oligofectamine (Life Technologies, Paisley, UK) in accordance with the manufacturer's protocols. At 4 h after transfection, DMEM supplemented with 20% FBS was added and incubated for 48 h.

We also prepared and assayed cell lysates for specific gene silencing by a western blotting protocol and harvested conditioned media for use in the functional assays described in the following.

### 2.6. Protein Extraction and Western Blot

Cells of both lines were respectively washed with PBS, and we extracted the protein from the cells with a triple-detergent lysis buffer, i.e., 0.1 M Tris-HCl, pH 7.4; 0.15 M NaCl, 1% (v/v) Triton X-100, 0.1% (v/v) Nonidet P-40, and 0.1% (w/v) sodium dodecyl sulfate (SDS) containing a complete mini protease inhibitor cocktail (Roche Diagnostics, Indianapolis, IN). We used the BCA protein assay kit (Thermo Fisher) to measure the protein concentrations. First, the total protein extracts (30 *μ*g) were separated using Mini-PROTEAN TGX precast gels (Bio-Rad, Hercules, CA) and transferred to membranes by the Trans-Blot Turbo Transfer System (Bio-Rad). After nonspecific protein binding was blocked with 5% bovine serum albumin (BSA), we incubated the membranes with antibodies to Tks5 (1 : 1,000, Proteintech), MMP-14 (1 : 2,000 Abcam), and *β*-actin (1 : 10,000, Sigma). Horseradish peroxidase (HRP)-conjugated secondary antibodies (Sigma) were diluted at 1 : 3,000. Each of the antibodies was diluted in blocking solution. Enhanced chemiluminescence was used to visualize the immunoreactive proteins with an ECL kit (Pierce Biotechnology, Rochford, IL). Densitometry was conducted with Adobe Photoshop.

### 2.7. Cell Migration Assay

The cancer cells' migration was evaluated in a 24-well modified Bowden chamber with 0.8 *μ*m-porepolycarbonate-membrane Transwell inserts (Corning, Corning, NY). For the generation of the myofibroblastic CAF-like phenotype, we stimulated NOFs with TGF-*β*1 (5 ng/mL) in SFM as described in [24] and collected the conditioned media as described above. Cancer cells (transfected with siRNA as described or mock-transfected) were trypsinised and resuspended in SFM at 5 × 10^5^ cells/mL, and we added 200 *μ*L of cell suspension to the migration chamber.

We then added NOF-CM or CAF-CM to the underside of the Transwell assay. After 24 h incubation, the unmigrated cells were removed from the inside of the migration chamber by swabbing. The cells that were adhering to the chamber's underside were fixed for 10 min in 100% (v/v) methanol. We then stained the cells with 0.1% (w/v) crystal violet and used a light microscope to count the cells. For the evaluation of cell migration, we counted the cells on the membrane's underside in 10 random images (at 200*x* magnification) per chamber.

### 2.8. Matrigel Invasion Assay

The invasive ability of SCC4 and H357 cells was assayed using Matrigel invasion chambers (BD Biosciences, Franklin Lakes, and NJ). First, the Matrigel chambers were re-hydrated in 500 *μ*L of SFM for 2 h, and then, the medium was removed from the insert. Cancer cells were trypsinised and resuspended in SFM at 5 × 10^5^ cells/mL and 200 *μ*L of cell suspension was added to the Matrigel invasion chamber. NOF-CM or CAF-CM was added to the underside of the chamber.

After 24-h incubation, we swabbed cells away from the inside of the invasion chamber's inner walls, and we fixed the cells that were adhering to the chamber's underside in 100% (v/v) methanol for 10 min. Next, 0.1% (w/v) crystal violet was applied to stain the cells, and we used light microscopy to count the stained cells. For the assessment of cell invasion, we counted the number of cells on the membrane's underside in 10 random images (200*x* magnifications) per chamber.

### 2.9. Immunohistochemical Staining

We obtained specimens from 60 patients with primary OSCC (30 males and 30 females) who had undergone surgical resection for their tumours at the Department of Oral and Maxillofacial Surgery at Kanazawa University Hospital (Kanazawa, Japan). The patients' written informed consent for their material to be used in this study was obtained, and the study was approved by the Kanazawa University Graduate School of Medical Science Ethics Committee (approval no. 2016-301(2072)).

The patients' ages ranged from 41 to 91 years (mean, 66.7 years). Their TNM categories were based on the UICC (Union for International Cancer Control) system, and their tumour differentiation grades were based on the criteria proposed by the World Health Organization (WHO). The mode of tumour invasion in each patient was assessed as described by Yamamoto and colleagues [25].

The immunohistochemical detection of Tks5 was performed with an anti-Tks5 rabbit polyclonal antibody pAb (Cell Signaling Technology, Danvers, MA). The immunohistochemical detection of *α*SMA was conducted with anti-*α*SMA rabbit pAb (Abcam, Cambridge, MA). Paraffin-embedded sections of tumour were deparaffinized and rehydrated, and then, endogenous peroxidase was blocked by the application of 0.3% hydrogen peroxide in methanol for 30 min. Nonspecific protein binding sites were blocked with goat serum for 10 min, and the sections were incubated with the primary antibodies overnight at 4°C.

Immunoreactive protein was detected by an Envision horse radish peroxidase (HRP) system (Dako, Kyoto, Japan). We used 1 mg/mL diaminobenzidine in the presence of 0.03% hydrogen peroxidase for the visualization of bound peroxidase, and we counterstained sections with hematoxylin. The staining specificity was confirmed with the use of nonimmune serum as a negative control instead of the primary antibody.

The expressions of Tks5 and *α*SMA at the invasive front were examined at 100*x* magnification. The expression score of each of these markers was calculated using the immunoreactive cell percentage and staining intensity. Each specimen received two scores, one assigned based on the percentage of positive cells (<10%: 1 point, 10%–50%: 2 points, and >50%:3 points) and the other based on the staining intensity (negative-to-weak: 1point, moderate: 2 points, and strong: 3 points). When the combined expression scores were ≥4 points, the tumour was classified as showing high Tks5 (Tks5+) or *α*SMA (*α*SMA+) expression. The expressions of Tks5 and *α*SMA were evaluated by two reviewers who were blinded to all details of the tumours. They assessed each marker's expression in relation to the following clinicopathological parameters: patient age and gender, T classification, N classification, stage, the degree of cell differentiation, and the mode of invasion.

### 2.10. Statistical Analyses

All of the experiments were conducted independently three or more times. Differences between pairs of groups were tested by the the independent samples *t*-test. We used the *χ*^2^-test to evaluate the relationships between the expression of Tks5 and *α*SMA and the above-described clinicopathological parameters.

The OSCC patients' 5-year survival rates were calculated by obtaining the Kaplan–Meier curves and then compared by the log-rank test. Factors that were identified as significant were then used in a Cox multivariate proportional hazard model for the determination of their prognostic values. Probability (*p*)-values were considered significant (^∗^*p* < 0.05, ^∗∗^*p* < 0.01, ^∗∗∗^*p* < 0.001). All of the statistical analyses were performed with SPSS version 16.0 software (SPSS, Chicago, IL).

## 3. Results

### 3.1. Culturing with Fibroblast-Derived Soluble Factors Increased the Motility and the Invasion of Cancer Cells

**Figure 1:**
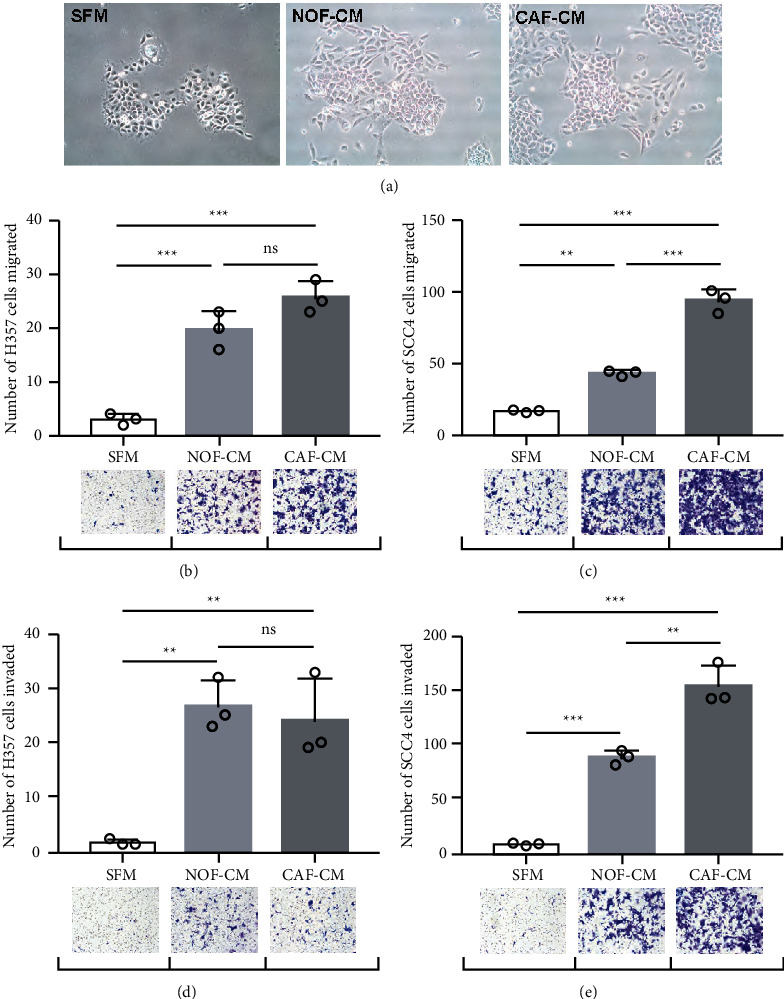
Indirect culture of oral squamous cell carcinoma (OSCC) cells with normal oral fibroblasts and cancer-associated fibroblasts (CAFs) promotes cancer cell migration and invasion. H357 and SCC4 cells were incubated (24 h) in conditioned medium (CM) collected from normal oral fibroblasts (NOFs), experimentally induced CAFs, or serum-free medium (SFM). (a) Images of cancer cells were captured by light microscopy. (b), (c) Cell migration was assessed by a Transwell system. (d), (e) Cell invasion through matrigel was examined. Representative images of migrated and invaded cells are shown under the respective experimental conditions, and the cells were quantified by counting cells on 10 randomly selected images. All experiments were conducted three times. The data are mean ± standard deviation (SD), ^∗^*p* <  0.05, ^∗∗^*p* <  0.01, ^∗∗∗^*p* <  0.001.

The exposure of SCC4 and H357 cells to conditioned medium derived from NOF (NOF-CM) or CAF (CAF-CM) produced the following results: some cells that had detached from pavement-like cell clusters formed filopodia and showed a more mesenchymal morphology, as assessed by light microscopy (Figure 1(a)). The migration of both the low-invasive H357 cells and the high-invasive SCC4 cells was significantly increased (*p* < 0.05) by the culturing with NOF-CN or CAF-CM (Figures 1(b) and 1(c)). Culture with CAF-CM induced significantly more migration of SCC4 cells compared to culture with NOF-CM (*p* < 0.05) (Figure 1(c)).

We observed the same pattern for invasion, with both the NOF-CM and the CAF-CM stimulating significantly elevated invasion of both cancer cell lines (Figures 1(d) and 1(e)).

### 3.2. Expression of the Markers of Invadopodia and Degradation of ECM

**Figure 2:**
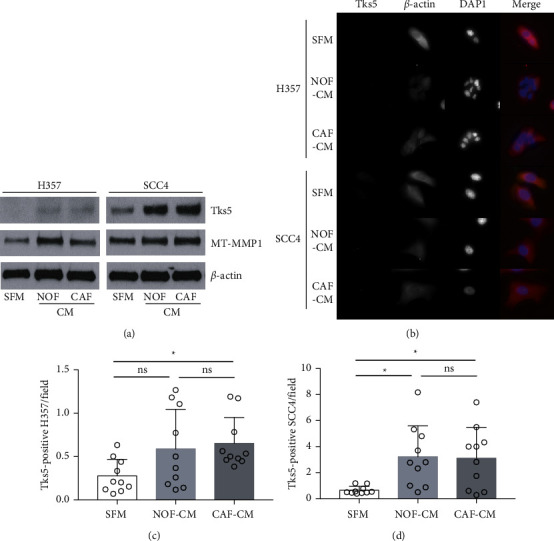
CAF-derived soluble factors promote the formation of Tks5-positive invadopodia formation in OSCC cells. H357 and SCC4 cells were incubated (24 h) in CM collected from NOFs, experimentally induced CAFs, or SFM. (a) The expression of Tks5 and MT-MMP1 protein in cancer cell lysates was analysed by western blotting. (b) The localization of Tks5 (indicative of the formation of invadopodia) was assessed by immunofluorescence. Merged images represent the immunofluorescence staining of Tks5 (green) with *β*-actin (red) and DAPI (blue) counterstaining. (c), (d): Three random areas were selected, and the number of Tks5-positive foci per cancer cell was determined using ImageJ. The data are mean ± SD, ^∗^*p* <  0.05, ^∗∗^*p* <  0.01, ^∗∗∗^*p* <  0.001.

To determine whether the changes in the OSCC cell lines' migration and invasion in response to fibroblast-derived cues may involve invadopodia, we first assessed the expression levels of the invadopodia-associated proteins Tks5 and membrane type 1-matrix metalloproteinase (MT1-MMP) by immunoblotting. The levels of both these proteins were significantly increased in the two cell lines on exposure to fibroblast-derived factors. Tks5 was barely detectable in H357 cells but was increased by culture with NOF-CM or CAF-CM compared to the culture with serum-free medium in both cell lines (Figure 2(a)). The MT1-MMP expression was increased in the cells cultured with NOF-CM and those cultured with CAF-CM (Figure 2(a)).

A punctate pattern of Tks5 expression was observed by immunofluorescence in both H357 and SCC4 cells, although a lower level of expression was observed in H357 cells (Figure 2(b)). When we cultured the two cell lines with CAF-CM, the expression of Tks5 was significantly increased in both cell lines; a significant increase was also observed in SCC4 cells in response to NOF-CM (Figures 2(c) and 2(d)).

### 3.3. Depletion of Tks5 Reduces Cancer Cell Invasion

**Figure 3:**
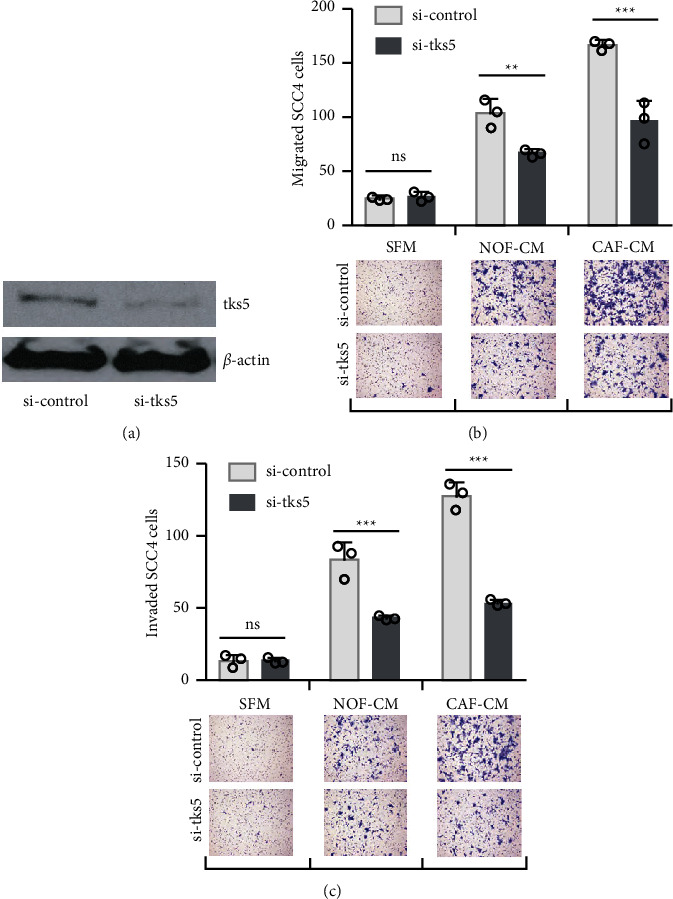
The depletion of Tks5 ameliorated the cancer cells' migration and invasion in response to CAF-derived cues. SCC4 cells were transfected with siRNA oligonucleotides targeting Tks5 (si-tks5) or nontargeting siRNA (si-control). (a) The expression of Tks5 was assessed by immunoblotting. (b), (c): The effect of NOF- and CAF-derived conditioned medium (NOF-CM and CAF-CM) on SCC4 migration and invasion, compared to SFM. The data are mean ± SD, ^∗^*p* <  0.05, ^∗∗^*p* <  0.01, ^∗∗∗^*p* <  0.001.

In order to analyze invadopodia formation's potential roles in the migration and the invasion of OSCC cells, we used siRNA to reduce the Tks5 expression in SCC4 cells, and we evaluated the effect of this on the response of the SCC4 to NOF- and CAF-derived factors. Western blotting revealed a significantly reduced level of Tks5 protein in the cells transfected with the siRNA specific to Tks5 compared to those transfected with the nontargeting control siRNA (Figure 3(a)). In response to NOF-CM and CAF-CM, the migrated cells transfected with Tks5 siRNA were significantly decreased in number compared to the control group (*p* < 0.01) (Figure 3(b)).

Our Transwell invasion assay also demonstrated that significantly fewer Tks5-depleted cells invaded through the Matrigel-coated chambers in response to NOF- and CAF-derived factors (*p* < 0.01) (Figure 3(c)).

### 3.4. Tks5 and *α*SMA Expressions Confer a Poor Prognosis

**Figure 4:**
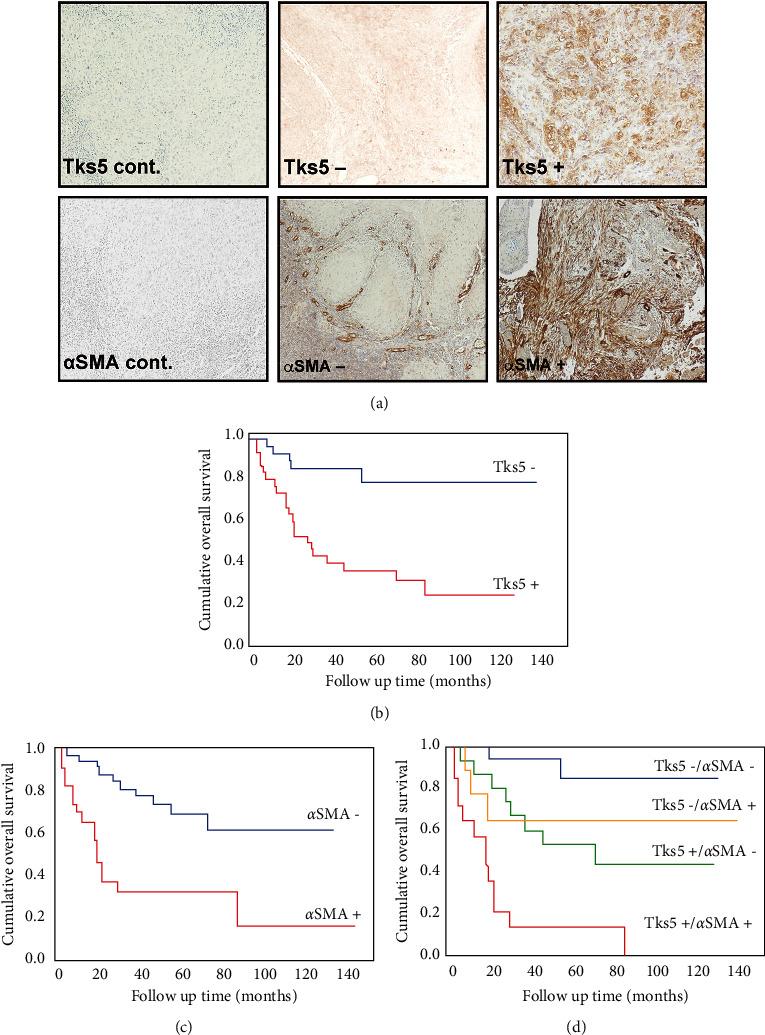
The expression of Tks5 and that of *α*SMA were associated with poor survival. Immunohistochemical detection of Tks5 and *α*SMA was carried out on a cohort of 60 patients with OSCC. The expression of the two markers was assessed by blinded reviewers and categorized as low (Tks5– or *α*SMA–) or high (Tks5+ or *α*SMA+). (a) Representative images of high- and low-expression cases. (b)–(d): The 5-year survival rates were calculated by the Kaplan–Meier method and compared by log-rank test.

Having observed changes in Tks5 protein expression in response to fibroblast-derived cues and functional effects of Tks5 depletion *in vitro*, we next examined the expressions of Tks5 and *α*SMA in OSCC tissue *ex vivo*. We observed Tks5 immunoreactivity predominantly in the cancer cells' cytoplasm. AlphaSMA immunoreactivity was observed mainly in the fibroblasts' cytoplasm, around tumour cells near the invasive front (Figure 4(a)). Significant correlations were identified between the Tks5 expression and the T classifications, N classifications, stage, and mode of invasion. We also observed significant correlations between the expression of *α*SMA and the N classification and stage (*p* < 0.05, respectively) (Table 1).

The OSCC patients' 5-year survival rate was 48.7% in the Tks5-positive group and 78.4% in the Tks5-negative group; the Tks5-positive group thus had significantly worse prognoses (*p* < 0.001) (Figure 4(b)). The *α*SMA-positive group's 5-year survival rate was 31.9% and the *α*SMA-negative group's was 69.1%; the *α*SMA-positive group had significantly worse prognoses (*p* < 0.001) (Figure 4(c)). Fifteen patients (51.4%) were positive for both Tks5 and *α*SMA. There were 15 Tks5-positive plus *α*SMA-negative patients, nine patients who were Tks5-negative plus *α*SMA-positive, and 21 patients who were negative for both Tks5 and *α*SMA. The patients who were positive for both Tks5 and *α*SMA had the poorest 5-year cumulative survival rate at 14.3% (Figure 4(d)). The univariate analysis showed that the T classifications, the mode of invasion, the expression of Tks5, and the expression of *α*SMA were significant prognostic factors. The multivariate analysis revealed that only the mode of invasion, Tks5 expression, and *α*SMA expression was independent prognostic factors (Table 2).

## 4. Discussion

An invasion of OSCC cells can lead to lymph node metastasis, disease recurrence, and mortality. One of the mechanisms by which cancer cells can invade surrounding stroma is via the formation of invadopodia, which are actin-rich and dynamic membrane protrusions at which focal ECM degradation occurs. Invasive cancer cells escape from the primary tumour by extending invadopodia into the surrounding ECM, in a process that is associated with matrix degradation and complex rearrangement of the actin cytoskeleton. Invadopodia contribute to transendothelial migration, which leads to the extravasation and metastasis of cancer cells. Tks5 has been reported to be necessary for the stability of invadopodia's precursor and for the degradation of the ECM [26]. Tks5 was also reported to have a critical role in the initiation of invadopodia formation in invasive cancer cells [27].

Cancer tissues are comprised of both cancer cells and various types of stromal cells such as fibroblasts, immune cells, and endothelial cells. The cancer-associated fibroblasts (CAFs) are frequently the most numerous cell types, and their role in cancer progression is significant [23]. CAFs promote cancer cells' proliferation and invasion by releasing mitogenic signals (e.g., growth factors and chemokines) and by modifying the extracellular matrix to support cells' dissemination and suppress immune responses. Attieh et al. reported that a tumour's microenvironment, especially CAFs, is important in the invasion of cancer cells [28]. In a study of OSCC cells, Li et al. demonstrated that CAFs have the ability to promote cancer progression [29]. However, the detailed mechanism of the interaction between cancer cells and CAFs in the progression of OSCC remains to be identified. In the present study, we focused on the formation of invadopodia, as they are indispensable for the invasion and migration of cancer cells. We also examined the effects of CAF-derived factors on the formation of the invadopodia in OSCC cells.

Interestingly, the results of this study indicate that both low-invasive OSCC-derived H357 cells and invasive SCC4 cells acquired increased migration and invasiveness after indirect culture with not only CAFs but also normal fibroblasts, in keeping with our recently reported findings [24]. Zeisberg et al. reported that epidermal growth factor (EGF), fibroblast growth factor 2 (FGF2), platelet-derived growth factor (PDGF), and transforming growth factor-*β* (TGF*β*) were each released by normal fibroblasts, and these factors affected the cancer cells' migration and invasion [30]. Other research studies also confirmed that fibroblasts that they isolated from fibrotic tissue maintained an activated phenotype and continued to secrete ECM-degrading enzymes, cytokines, and growth factors [15, 18, 31, 32]. It was also reported that extracellular vehicles released by normal fibroblasts affected the proliferation of colorectal cancer cells [33]. These results reinforce the concept that stromal fibroblasts in the tumour microenvironment are important and necessary components for cancer cells to migrate and invade, and that these fibroblasts positively influence the invasiveness of OSCC cells.

In the present study, metastatic OSCC-derived SCC4 cells expressed significantly higher levels of Tks5 compared to nonmetastatic H357 cells. The expression of Tks5 protein, but not the transcript, was increased in both OSCC cell lines upon culture with NOF-CM or CAF-CM, especially prominent in the SCC4 cells. This outcome suggests that (i) the expression of Tks5 is regulated by factors secreted by CAFs in the tumour microenvironment, and (ii) cancer cell invasion and metastasis are promoted by the any secretions from CAFs. Further research is necessary to determine the mechanism that underlies the observed increase in the expression of Tks5.

The focal degradation of the ECM is a key step in cancer cells' invasion into the surrounding mesenchyme. MT1-MMP (which is also known as MMP14) is a proteinase located at cancer cells' membranes, and it is overexpressed in aggressive tumours [34]. MT1-MMP activates MMP2 and MMP9 and leads to a degradation of the ECM [35]. It was also reported that MT1-MMP accumulates in invadopodia [36]. We observed that the MT1-MMP expression in two OSCC cell lines was increased by culturing with NOF-CM and with CAF-CM, suggesting that one mechanism by which fibroblasts stimulate cancer cell invasion may be by promoting MT1-MMP expression.

Inhibition of the functioning of Tks5 reduces gelatin degradation and the invasiveness of breast cancer and melanoma cells [12]. Here, we utilised siRNA to examine whether Tks5 is necessary for the migration and invasion of OSCC cells. We observed that the abrogation of Tks5 expression in both the SCC4 and H357 cell lines reduced the cell migration and the cell's invasion ability in response to fibroblast-derived CM (both NOF and CAF). These observations suggested that Tks5 has a role in the dissemination of OSCC in response to microenvironmental cues.

Tks5 overexpression is associated with both metastasis and poor prognosis in several types of cancers [37, 38]. Moreover, in an immunohistochemical study of a murine model of peritoneal dissemination, prominent Tks5 expression was identified in peritoneal mesothelial cells at the invasion front [39]. We also observed readily detectable levels of Tks5 expression at the invasive front of OSCC cells, and the overexpression of Tks5 was significantly associated with both the tumour stage and the patients' prognoses. These results suggested that the expression of Tks5 at the invasive front of a tumour is significantly correlated with the tumour spread and progression in OSCC. In addition, a high expression of *α*SMA, a marker of CAFs, was associated with a poor prognosis, in agreement with an earlier analysis [40]. High levels of both Tks5 and *α*SMA conferred the worst prognosis in the present study's OSCC patients, providing *ex vivo* support for the *in vitro* evidence presented here of *α*SMA-positive CAFs promoting invadopodia-mediated cancer cell invasion.

In conclusion, we obtained evidence that the formation of invadopodia and degradation of ECM in OSCC are influenced by an indirect interaction between OSCC cells and the stromal fibroblasts. We also observed that by inhibiting Tks5, a key protein in invadopodia formation, both the migration and invasion of OSCC cells can be suppressed. Taken together, the results of this study suggest that interactions between the tumour microenvironment and cancer cells influence the formation of invadopodia, and this knowledge may help identify novel opportunities for therapeutic interventions in OSCC.

## Figures and Tables

**Table 1:** Clinicopathological parameters in relation to Tks5 or *α*SMA expressions.

	*n*	Tks5		*p* value	*α*SMA		*p* value
		+	−		+	−	
*Age*							
<65	26	12	14	0.602	8	18	0.202
65<	34	18	16		16	18	
*Gender*							
Male	30	15	15	1.000	12	18	1.000
Female	30	15	15		12	18	
*T classification*							
T1	10	3	7	0.036	2	8	0.334
T2	35	15	20		14	21	
T3	3	2	1		1	2	
T4	12	10	2		7	5	
*N classification*							
N0	41	17	24	0.042	11	30	0.008
N1	11	6	5		8	3	
N2, N3	8	7	1		5	3	
*Stage*							
I	9	2	7	0.035	2	7	0.043
II	24	10	14		6	18	
III	10	5	5		5	5	
IV	17	13	4		11	6	
*Cell differentiation*							
Well	28	14	14	0.747	8	20	0.071
Moderate	22	10	12		9	13	
Poor	10	6	4		7	3	
*Mode of invasion*							
1	6	0	6	0.048	0	6	0.185
2	8	3	5		2	6	
3	24	13	11		11	13	
4C	11	7	4		6	5	
4D	11	7	4		5	6	

**Table 2:** Univariate and multivariate analyses for clinicopathological parameters, Tks5, and *α*SMA expression in relation to overall survival for 60 patients with OSCC.

Variables	Clinical groups	Survivors (*n* = 34)	Nonsurvivors (*n* = 26)	Log rank		Cox regression *p* value	Risk ratio
				Χ^2^	*p* value		
T category	T1-2/T3-4	28/6	17/9	4.125	0.0422	0.0577	
N category	N−/N+	26/8	15/11	3.401	0.0652		
Stage	S1-2/S3-4	22/12	11/15	0.482	0.4877		
Cell differentiation	Well/moderate and poor	16/16	14/14	0.254	0.6141		
Mode of invasion	1, 2, 3/4C and 4D	25/9	13/13	9.381	0.0022	0.0107	3.028
Tks5	−/+	25/9	5/21	12.454	0.0004	0.0056	3.652
*α*SMA	−/+	26/8	10/16	12.370	0.0004	0.0025	3.403

## Data Availability

The data that support the findings of this study are available from the corresponding author upon reasonable request.
